# Automated detection and removal of flat line segments and large amplitude fluctuations in neonatal electroencephalography

**DOI:** 10.7717/peerj.13734

**Published:** 2022-07-12

**Authors:** Gabriella Tamburro, Katrien Jansen, Katrien Lemmens, Anneleen Dereymaeker, Gunnar Naulaers, Maarten De Vos, Silvia Comani

**Affiliations:** 1 Department of Neuroscience, Imaging and Clinical Sciences, University “G. d’Annunzio” of Chieti-Pescara, Chieti, Italy; 2 BIND – Behavioral Imaging and Neural Dynamics Center, University “G. d’Annunzio” of Chieti-Pescara, Chieti, Italy; 3 Department of Development and Regeneration, UZ Leuven, Leuven, Belgium; 4 Department of Electrical Engineering (ESAT), STADIUS Center for Dynamical Systems, Signal Processing and Data Analytics, KU Leuven, Leuven, Belgium

**Keywords:** Neonatal EEG, Automated artifact detection, Flat lines, Large amplitude fluctuations

## Abstract

**Background:**

Artefact removal in neonatal electroencephalography (EEG) by visual inspection generally depends on the expertise of the operator, is time consuming and is not a consistent pre-processing step to the pipeline for the automated EEG analysis. Therefore, there is the need for the automated detection and removal of artefacts in neonatal EEG, especially of distinct and predominant artefacts such as flat line segments (mainly caused by instrumental error where contact between electrodes and head box is lost) and large amplitude fluctuations (related to neonatal movements).

**Method:**

A threshold-based algorithm for the automated detection and removal of flat line segments and large amplitude fluctuations in neonatal EEG of infants at term-equivalent age is developed. The algorithm applies thresholds to the absolute second difference, absolute amplitude, absolute first difference and the ratio between the frequency content above 50 Hz and the frequency content across all frequencies.

**Results:**

The algorithm reaches a median accuracy of 0.91, a median hit rate of 0.91 and a median false discovery rate of 0.37. Also, a significant improvement (≈10%) in the performance of a four-stage sleep classifier is observed after artefact removal with the proposed algorithm as compared to before its application.

**Significance:**

An automated artefact removal method contributes to the pipeline of automated EEG analysis. The proposed algorithm has shown to have good performance and to be effective in neonatal EEG applications.

## Introduction

Electroencephalography (EEG) is a fundamental tool in the Neonatal Intensive Care Unit (NICU) for the real-time monitoring of the cerebral function of preterm and sick term neonates. Automated analysis of neonatal EEG has proven its usefulness in applications such as seizure detection (Abdelhameed & Bayoumi, 2019; Ansari et al., 2019; Becker et al., 2021), sleep classification (Dereymaeker et al., 2017; Pillay et al., 2018; Ansari et al., 2020), microstate analysis (Khazaei et al., 2021), stress quantification (Lavanga et al., 2020), outcome prediction (Duffy & Als, 2012; Peters et al., 2013; Lavanga et al., 2021) and functional brain age estimation (Lavanga et al., 2018; Pillay et al., 2020). Consequently, these analyses aim to detect those neonates that are most vulnerable and to improve clinical care in the NICU.

Unfortunately, EEG does not only include cerebral activity but also likely records several artefacts which do not originate from the brain and can thus obstruct the correct interpretation and further analysis of the EEG. Generally, artefacts are classified in two groups: (1) physiological artefacts that originate from other organs of the human body, *e.g*., eye movements, eye blinks, cardiac interference and muscular artefacts, and (2) non-physiological artefacts that originate from outside the human body, *e.g*., gross body movements, faulty electrodes, power line interference and electrode impedance. Although neonatal EEG recordings are generally quite long, artefactual segments are typically removed after visual inspection by an expert before applying automated EEG analysis. The effectiveness and reliability of this procedure depends on the expertise of the operator, is in any case time consuming and generates a non-automated pre-processing step to the pipeline for the automated EEG analysis. Therefore, it is recommended to detect and/or remove artefacts from neonatal EEG recordings in an automated way.

Various algorithms for the automated detection and/or removal of neonatal EEG artefacts have been introduced in the literature that differ in complexity, usefulness, generalizability, and performance. In addition, some algorithms focus on one specific type of artefact, whereas others aim to detect a broader range of artefacts. As such, Bhattacharyya et al. (2019) detected only electrical cortical stimulation artefacts by using spatial filtering in combination with tunable-Q wavelet transform, whereas Navarro et al. (2015) used complete ensemble empirical mode decomposition followed by adaptive filtering to detect only ECG artefacts. In contrast, several artefacts such as eye blinks, movement artefacts, muscle noise, cardiac signals and power line interference are detected with a finite impulse response filter by Abbasi et al. (2019). Another common approach to detect a broad range of artefacts is to use several features in a classification or clustering model. This has been done with support vector machines by Bhattacharyya et al. (2013) reaching an accuracy of 75% and by Stevenson et al. (2014) reporting 100% and 89% accuracy for respectively major and minor artefacts (distinction based on amplitude) and with semi-supervised Gaussian mixture models by Kauppila, Vanhatalo & Stevenson (2017) obtaining 95% accuracy. Neural networks have also been used for the detection of artefacts in neonatal EEG. For instance, Schetinin & Schult (2004) used a multi-layer polynomial neural network in combination with a decision tree reaching an accuracy of 73.5%, whereas Webb et al. (2021) used a residual deep neural network obtaining 87% accuracy. These accuracies were calculated by using artefact annotations. However, the performance of artefact detection methods can also be assessed by considering the change in performance of an application that is employed once on the original neonatal EEG recordings and once after using an artefact detection method. For instance, De Vos et al. (2011) verified that a seizure detector performed better after removal of ECG, pulsation and respiration artefacts using Independent Component Analysis (ICA), reporting a significant decrease in false positive seizure detections. Also, Khlif et al. (2010) described a significant improvement in the performance of a seizure detector after removal of several artefact types with frequency filtering and matching pursuit decomposition. Another application is quiet sleep detection for which sensitivity, specificity, misclassification factor and area under the curve were significantly improved when artefacts were removed with the artefact subspace reconstruction method, as reported by Dereymaeker et al. (2017).

**Figure 1:**
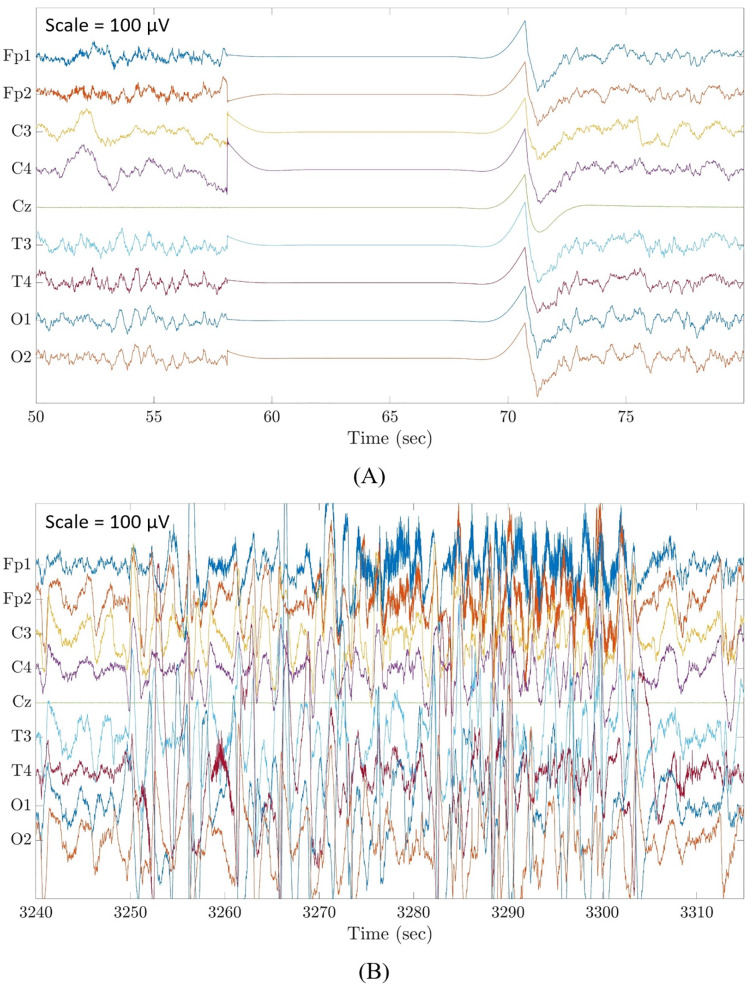
Examples of flat line segment and large amplitude fluctuations in neonatal multichannel EEG recordings. (A) Example of a flat line segment in patient 3 of Dataset 3. (B) Example of large amplitude fluctuations in patient 4 of Dataset 3. Scale is 100 μV.

Although there are already many automated artefact detection methods available in the literature, less attention was given specifically to flat line segments caused by an instrumental error where contact between electrodes and head box is temporary lost (Fig. 1A), and large amplitude fluctuations that are most often known as movement artefacts (Fig. 1B). These are distinct and predominant artefacts commonly affecting neonatal EEG recordings which must be removed before any algorithm for the analysis of EEG recordings is applied. Zima et al. (2012) focused on the detection of short-duration high-amplitude artefacts by using ICA and wavelet denoising. Other authors applied a threshold to the EEG amplitudes, as done by Costa (2017), who used a threshold of 100 μV, and by Sharif, Alsibai & Kushnerenko (2017), who applied a threshold of 200 μV. Alternatively, other scientists applied a threshold to the mean EEG amplitudes in a given epoch, as performed by Kauppila, Vanhatalo & Stevenson (2017) with a threshold of 200 μV and by Stevenson et al. (2012) with a threshold of 120 μV. Very recently, Kumaravel et al. (2022) proposed an artefact removal pipeline for the denoising of infant and newborn EEG recordings. Their method focused on the detection of channels with high-amplitude artefacts, flat or weakly responsive channels and on bad EEG segments containing amplitude jumps and paroxysmal artefacts. The method was tailored for high-density EEG recordings (125 channels) and was validated by assessing the statistical significance of the neural responses to a visual stimulus using the power spectral density and/or the event related potentials calculated on the cleaned EEG data.

Most of these threshold-based methods did not focus on the detection of large amplitude fluctuations and the algorithms developed for denoising the EEG signals were generally considered as a pre-processing step for EEG analytical applications without calculating the statistical performance of the artefact removal step. Therefore, there is a need for a correctly validated algorithm for the automated detection and removal of flat line segments and large amplitude fluctuations without imposing any limitation to the duration of the artefactual segments.

The current study proposes a threshold-based algorithm for the automated detection and removal of flat line segments and large amplitude fluctuations from neonatal EEG recordings. Several novel aspects can be identified with respect to already available methods: (1) the thresholds used for detecting flat lines and large amplitude fluctuations were tailored to the term-equivalent age of the neonate because it has an influence on the amplitude of the EEG signals; (2) the automated detection of flat line segments and large amplitude fluctuations was performed using an easy approach that has a low computational load; (3) the proposed algorithm was not only validated by assessing the changes of the performance of a neonatal EEG application after having applied our algorithm–as most existing methods do–but it was also tested in terms of statistical performance, differently from the majority of existing methods developed to pre-process the neonatal EEG.

The structure of the remainder of the paper is as follows: The Materials & Methods section describes the datasets used in the current study and explains the different steps of the threshold-based algorithm to detect and remove flat line segments and large amplitude fluctuations in an automated way. At the end of this section, the two validation approaches are explained: (1) validation based on statistical performance measures including parameter selection and (2) validation based on the change in performance of an EEG application. Then, the results are presented, followed by the discussion of these results. Finally, the paper will be concluded.

## Materials and Methods

### Datasets

The multi-channel EEG recordings of infants at term-equivalent age used for the present study were selected from three different datasets recorded at different sites. The selected EEG recordings did not include any seizures or any other signs of pathological conditions. The reason for using different datasets was to test the proposed algorithm on EEG signals recorded with different EEG systems and with a different number of electrodes. No EEG recording from these datasets originally included annotations for flat lines and large amplitude fluctuations. The main information on the three datasets is summarized in Table 1.

**Table 1:** Summary of the relevant information on the three datasets. For each dataset, the table includes: the number of EEG recordings; the number of EEG recordings selected for the present study; the number of EEG channels in the recordings; the sampling frequency of the EEG recordings; the neonatal postmenstrual age (PMA) at the time of recording; the duration of the EEG recordings; the number of EEG recordings contaminated by flat line segments; the number of EEG recordings contaminated by large amplitude fluctuations and availability of annotations for flat line segments and large amplitude fluctuations. Data for PMA and recording duration are provided as median [interquartile range].

	Dataset 1	Dataset 2	Dataset 3
Number of EEG recordings	79	136	16
Number of selected EEG recordings	22	9	16
Number of EEG channels	19	8	9
Sampling frequency (Hz)	256	250	250
PMA (weeks)	41.00 [39.50–42.50]	39.07 [37.00–40.71]	40.36 [39.93–41.50]
Duration of EEG recording (minutes)	74.92 [62.98–83.30]	300.00 [300.00–300.00]	1,004.59 [890.21–1050.71]
Number of EEG recordings contaminated by flat lines	20	0	5
Number of EEG recordings contaminated by large amplitude fluctuations	22	8	16
Annotations	No	No	No

#### Dataset 1

This public dataset (freely available at online repository, Zenodo, https://zenodo.org/record/2547147#.YsXck3bMJPZ) was recorded at the Helsinki University Hospital (Finland) (Stevenson et al., 2019) and contains EEG recordings registered with a NicOne EEG amplifier (Natus, San Carlos, CA, USA) and 19 channel EEG caps (sintered Ag/AgCl electrodes; Waveguard, ANT Neuro, Freiburg, Germany, Cz as reference electrode) following the international 10-20 system (Stevenson et al., 2019).

#### Dataset 2

This dataset originates from the Resilience Study performed at the NICU of the University Hospitals Leuven (Belgium). The EEG data were recorded with the Brain RT EEG recording system (OSG BVBA, Kontich, Belgium) using nine electrodes with Cz as reference electrode, in accordance with the international 10-20 system.

#### Dataset 3

This dataset was also recorded at the NICU of the University Hospitals Leuven (Belgium) with the same recording system and electrodes as Dataset 2. These EEG recordings had been used to develop a four-stage sleep classifier and annotated to identify the four sleep stages (Ansari et al., 2020).

Datasets 1 and 2 were used for training and testing the algorithm. The EEG segments automatically detected as artefactual were checked by visual inspection by two independent, non-clinical investigators (LS and GT). Dataset 3 was used to validate the proposed algorithm by applying it before a four sleep stage classifier. To this end, for each EEG recording a randomly selected 20-min segment was selected to represent the entire recording and annotated by a clinician (KL) for large amplitude fluctuations. On average, these annotated intervals corresponded to 20.43% of the duration of the selected 20-min EEG segments. For all EEG recordings selected for this study, flat lines were visually checked by two independent, non-clinical experts (LS and GT).

### Algorithm

**Figure 2:**
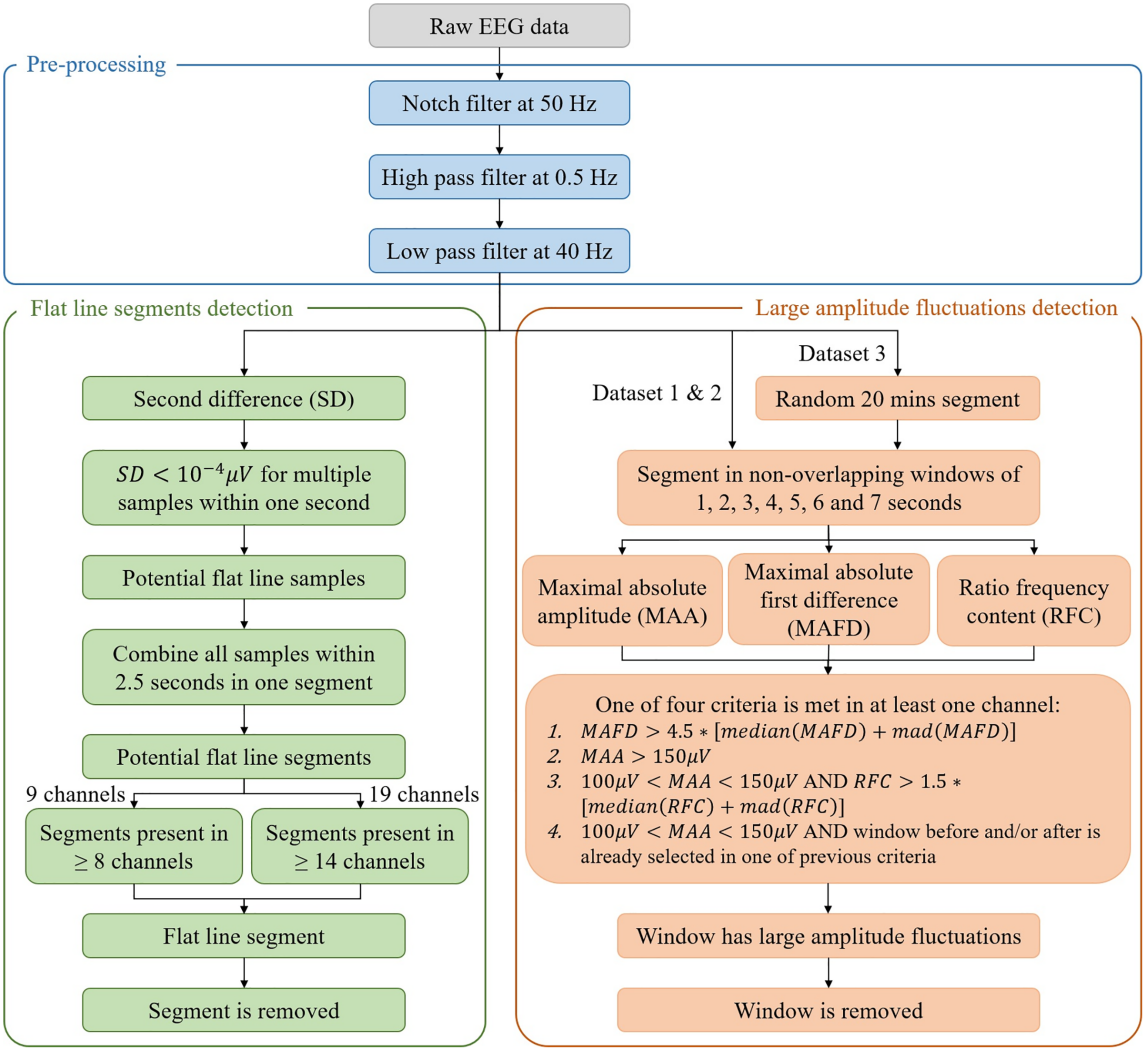
Flowchart of the algorithm for the automated detection of flat line segments and large amplitude fluctuations in neonatal multichannel EEG recordings. In the figure, mad stands for median absolute deviation.

A complete overview of the algorithm is given in Fig. 2.

#### Pre-processing

All datasets used for this study included EEG recordings that did not show any bad channels (*i.e*., channels being flat or showing large amplitude fluctuations during the entire recording). When an EEG recording has bad channels, it would be necessary to remove them and, eventually, replace them with EEG signals reconstructed by interpolating the signals recorded by the neighboring channels.

Given that the EEG recordings of Dataset 1 were already preprocessed with a notch filter at 50 Hz to remove power line interference, we applied a notch filter at 50 Hz also to the EEG recordings of Datasets 2 and 3 to conform the preprocessing of all datasets. The EEG recordings were then band-pass filtered between 0.5 and 40 Hz.

**Figure 3:**
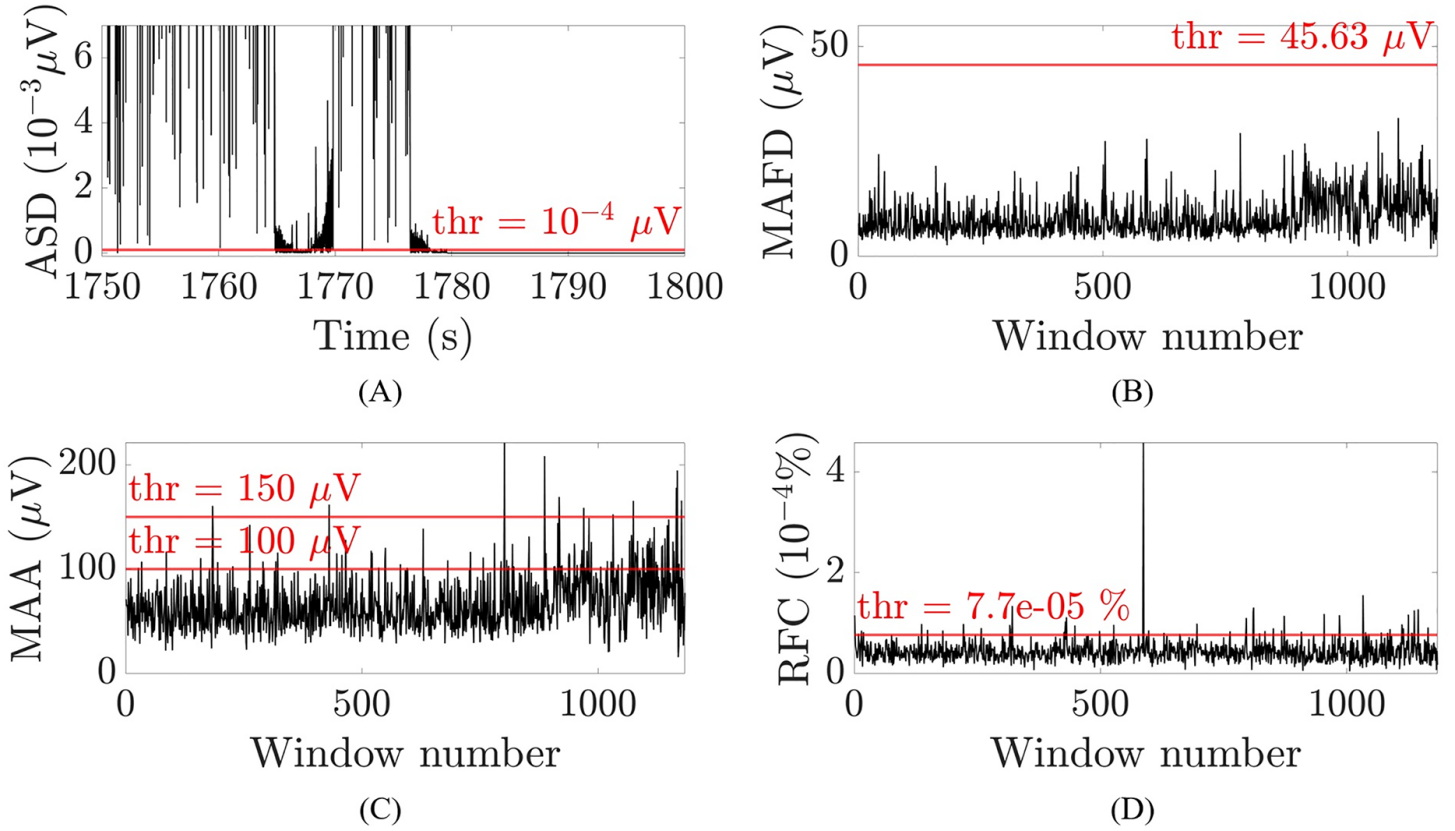
Examples of the thresholds used for the selection of the artefactual segments for one patient and channel O2. (A) Absolute second difference (ASD) in 10^−3^ μV (random 50 s segment, zoomed-in). (B) Maximal absolute first difference (MAFD) in μV for window length of 3 s. (C) Maximal absolute amplitude (MAA) in μV for window length of 3 s. (D) Ratio of frequency content (RFC) in 10^−4^% for window length of 3 s. The red horizontal lines represent the threshold levels.

*Flat line segments*. To detect flat line segments, the second difference across the whole EEG acquisition with N samples was computed for each channel, *i.e*.,

(1) $$z\left( n \right) = y\left( {n + 1} \right) - y\left( n \right)\,\rm {where} \,y\left( n \right) = x\left( {n + 1} \right) - x\left( n \right),$$

where $z$ is the second difference, $y$ is the first difference, $x$ is the EEG amplitude at a given time instant $n$ and $n = 1 \ldots \left( {N - 2} \right)$. A threshold of ${10^{ - 4}}\; \mu V$, selected to optimize the performance of the algorithm, was then applied to the absolute value of the second difference. Whenever multiple samples $z\left( n \right)$ within one second (*i.e*., individual samples which were not necessarily consecutive) were smaller than the threshold, these samples $z\left( n \right)$ were suspected to belong to a flat line segment and selected to be removed. Figure 3A illustrates an example for one patient. To avoid missing samples that were slightly higher than the threshold but part of the flat line segment, selected samples were combined into one interval when they were within 2.5 s apart from each other. These intervals were identified as flat line segments and removed only if they were selected in at least 14 out of 19 channels (Dataset 1) or in at least seven out of nine channels (Datasets 2 and 3), which corresponds to about the 75% of the channels affected by the flat line. To guarantee that the whole segment showing flat line was removed, two additional segments of 1 or 3 s were removed before and after the flat line interval, depending on whether it was shorter or longer than 5 s. Adding 1 or 3 s to the flat line segment to be removed was possible because neonatal EEG recordings are typically very long (lasting for tens of hours or more) and adding few seconds to the segment identified as flat line would not compromise the availability of sufficiently long clean EEG segments for further analysis. Furthermore, flat lines are often preceded/followed by sharp deflections of the signal. Adding 1 to 3 s to the detected flat line ensures that all the artefactual segment is removed.

#### Large amplitude fluctuations

For the detection of large amplitude fluctuations, the EEG acquisition is segmented in non-overlapping windows for which different durations are tested (*i.e*., 1, 2, 3, 4, 5, 6 and 7 s). For each window and each channel, the maximal absolute first difference (MAFD), the maximal absolute amplitude (MAA) and the ratio between the frequency content above 50 Hz and the frequency content across all frequencies (RFC) are computed:

(2) $$MAFD = {\rm max}\ \left( {\left| {x\left( {n + 1} \right) - x\left( n \right)} \right|} \right)$$

(3) $$MAA = {\rm max}\ \left( {\left| {x\left( {\Delta t} \right)} \right|} \right)$$

(4) $$RFC = \; \displaystyle{{P(f > 50\; Hz)} \over {P\left( f \right)}}$$

where $x$ is the EEG signal, $\Delta t$ is the window duration, $n = 1 \ldots {f_s}\Delta t$, ${f_s}$ is the sampling frequency, $P$ is the Welch’s power spectral density estimate with Hamming window with 50% overlap, and $f$ is the frequency in the range $\left[ {0,\textstyle{{{f_s}} \over 2}} \right]$ (Mulkey et al., 2019; Vesoulis et al., 2020; Rayson et al., 2022). The MAFD was calculated to detect segments that have an abrupt change in amplitude, whereas the RFC was used to recognize muscular artefacts (André et al., 2010) because they often occur together with movement artefacts. Concerning the MAA, a physiological threshold of 100 μV (PT100) was applied above which no cortical activity is seen in the EEG recordings of infants at term-equivalent age (André et al., 2010). Nevertheless, a distinction was made between windows with MAA higher than 150 μV (PT150), that are certainly not related to cortical activity and can thus be removed, and the windows with MAA between PT100 and PT150 which are treated as a doubtful case. The latter windows were removed only when their RFC was above a given threshold indicating the presence of muscular artefacts or when the window before and/or after was selected to be removed. Consequently, a window was removed if and only if one of the following four criteria was met in at least one channel:

1.
$MAFD > 4.5\cdot \left[ {median\left( {MAFD} \right) + median\; absolute\; deviation\left( {MAFD} \right)} \right]$ (channel-specific threshold);
2.
$MAA > PT150$;
3.
$PT100 < MAA < PT150$ AND $RFC \gt 1.5\cdot \left[ {median\left( {RFC} \right) + median\; absolute\; deviation  \left( {RFC} \right)} \right]$ (channel-specific threshold);
4.
$PT100 < MAA < PT150$ AND the window before and/or after was already selected in one of the previous criteria.

The channel-specific thresholds in the first and third criterion were chosen by trial and error, *i.e*., by visually checking whether the selected segments in Datasets 1 and 2 were selected correctly. The abovementioned measures and their thresholds are illustrated in Figs. 3B–3D for one patient for channel O2 and a window of 3 s.

Consecutive windows selected to be removed were combined into one interval, and two segments selected to be removed that were within 6 s from each other were also combined into one interval to guarantee that all large amplitude fluctuations were removed.

### Parameter selection and statistics-based validation

The large amplitude fluctuation annotations of Dataset 3 were used to assess the performance of the algorithm by means of three statistical measures based on the confusion matrix:

1. The accuracy, *i.e*., the percentage of the signal that was classified correctly: $\displaystyle{{TP + TN} \over {TP + FP + FN + TN}}$;
2. The hit rate (HR), *i.e*., the proportion of the signal annotated as artefact that was detected as artefact: $\displaystyle{{TP} \over {TP + FN}}$;
3. The false discovery rate (FDR), *i.e*., the proportion of the signal detected as artefact that was annotated as non-artefact: $\displaystyle{{FP} \over {TP + FP}}$.

where: if both the algorithm and the expert detected a large amplitude fluctuation, the outcome was designated as true positive (TP); if both the algorithm and the expert detected no large amplitude fluctuation, the outcome was designated as true negative (TN); if the algorithm detected a large amplitude fluctuation that was not labelled by the expert, the outcome was designated as false positive (FP); if the algorithm did not detect a large amplitude fluctuation that was labelled by the expert, the outcome was designated as false negative (FN).

Differences in these performance metrics between the different window durations were statistically tested with the nonparametric Kruskal-Wallis test followed by Bonferroni correction for pairwise comparisons to select the window duration that yields the best performance of the algorithm. Thereafter, the effect size was computed for each performance metric with the use of epsilon squared ( $E_R^2$) for Kruskal-Wallis test (Tomczak & Tomczak, 2014; Torres-Gordillo, Melero-Aguilar & García-Jiménez, 2020). Epsilon squared is a coefficient that describes the strength of the effect size and varies between 0 (negligible effect) and 1 (very strong effect) (Castillo et al., 2020). The abovementioned performance metrics were applied only to Dataset 3 to assess the performance of the algorithm in the detection of large amplitude fluctuations.

### Application-based validation

As mentioned in Datasets section, Dataset 3 was used by Ansari et al. (2020) to develop a convolutional neural network to perform 2-and 4-class sleep classification in neonates. The network takes as input 30-s EEG segments and outputs a probability for each sleep stage for each 30 s interval of the EEG recording. The sleep stage with the highest probability will be considered as the actual sleep stage of the infant.

This EEG application was used to further validate the performance of the proposed algorithm to detect and remove flat line segments and large amplitude fluctuations. We used the window duration selected in the previous section as the one yielding the best combination of values for accuracy, HR and FDR. More specifically, the performance of the four-stage sleep classifier was assessed on the entire neonatal EEG recordings before and after removing flat line segments and large amplitude fluctuations with our algorithm. The purpose of this validation procedure is to demonstrate that the automated detection and removal of flat line segments and large amplitude fluctuations improves the performance of the sleep classifier.

Three statistical performance measures based on the extended confusion matrix in Table 2 were calculated to compare the performance of the sleep classifier before and after applying the proposed algorithm on the EEG recordings of Dataset 3:

**Table 2:** Extended confusion matrix. The extended confusion matrix is used to test the performance of the four-stage sleep classifier before and after applying the proposed algorithm to remove flat line segments and large amplitude fluctuations. A, B, C and D are used for illustrative purposes and correspond to the four sleep stages.

		Predicted sleep stage				Sum	Sensitivity (one *vs*. all)
LVI	ASI	HVS	TA				
True sleep stage	**LVI**	AA	AB	AC	AD	$A\sim$	$\displaystyle{{AA} \over {A\sim}}$
	**ASI**	BA	BB	BC	BD	$B\sim$	$\displaystyle{{BB} \over {B\sim}}$
	**HVS**	CA	CB	CC	CD	$C\sim$	$\displaystyle{{CC} \over {C\sim}}$
	**TA**	DA	DB	DC	DD	$D\sim$	$\displaystyle{{DD} \over {D\sim}}$
Sum		$\sim A$	$\sim B$	$\sim C$	$\sim D$		**Accuracy**
Precision (one *vs*. all)		$\displaystyle{{AA} \over {\sim A}}$	$\displaystyle{{BB} \over {\sim B}}$	$\displaystyle{{CC} \over {\sim C}}$	$\displaystyle{{DD} \over {\sim D}}$		$\displaystyle{{AA + BB + CC + DD} \over {all\; entries}}$

**Note:**

LVI, Low Voltage Irregular; ASI, Active Sleep I; HSV, High Voltage Slow; TA, Tracé Alternant.

1. The overall accuracy, *i.e*., the percentage of the signal correctly classified: $\displaystyle{{AA + BB + CC + DD} \over {all\; entries}}$;
2. The precision, calculated as one class *vs*. all classes, *i.e*., the proportion of the signal predicted to be the considered class that was also annotated as that class: *e.g*., $\displaystyle{{AA} \over {\sim A}}$ for the predicted class LVI. The mean precision across all classes was then calculated as: $\left( {\displaystyle{{AA} \over {\sim A}} + \displaystyle{{BB} \over {\sim B}} + \displaystyle{{CC} \over {\sim C}} + \displaystyle{{DD} \over {\sim D}}} \right)/4$;
3. The sensitivity, calculated as one class *vs*. all classes, *i.e*., the proportion of the signal annotated as the considered class that was also predicted to be that class: *e.g*., $\displaystyle{{AA} \over {A\sim}}$ for the true class LVI. The mean sensitivity across all classes was then calculated as: $\left( {\displaystyle{{AA} \over {A\sim}} + \displaystyle{{BB} \over {B\sim}} + \displaystyle{{CC} \over {C\sim}} + \displaystyle{{DD} \over {D\sim}}} \right)/4$.

In addition, the confidence of the sleep classifier was calculated before and after applying the proposed algorithm as the difference between the probabilities of the sleep stages with the two highest probabilities. This metric takes into account that the second-highest probability could be only slightly lower than the probability of the winning class such that there is low confidence that the winning class is the right class. The mean confidence across all 30 s epochs was then considered.

Differences in the confidence of the classifier and the three statistical performance metrics before and after removing flat line segments and large amplitude fluctuations were statistically tested with the nonparametric Wilcoxon rank sum test. After that, the effect size ( ${\eta ^2}$) was calculated for the three performance metrics because the sample sizes were relatively small. This effect size indicates the amount of the variance in the dependent variable that is explained by the independent variable, and varies between 0 (negligible effect) and 1 (very strong effect) (Tomczak & Tomczak, 2014). EEG processing and statistical analysis was performed in MATLAB software (Version R2020a; MathWorks, Natick, MA, USA).

## Results

### Parameter selection and statistics-based validation

#### Flat line segments

The proposed algorithm removed on average 2.00% of the recordings of Dataset 1 as flat line segments. The average removal percentage of Dataset 3 was 0.06%, whereas for Dataset 2 it was 0% because no flat line segments were present.

Although no annotations for flat line segments were available for any dataset, it was verified by visual inspection that all flat lines were detected by the algorithm for both Dataset 1 and 3, hence reaching an accuracy of 100%.

#### Large amplitude fluctuations

An average of 41.12%, 23.89% and 47.02% of the recordings of Datasets 1, 2 and 3, respectively, across all window durations, was removed as they were detected as affected by large amplitude fluctuations. This outcome indicates a high prevalence of this type of artefact. The following results were obtained by using the 20-min annotated segments of Dataset 3.

A significant strong difference ( ${\chi ^2} = 42.22$, degrees of freedom = 111, $p = 1.66\cdot {10^{ - 7}}$, $E_R^2 = 0.38$) in accuracy between the different window durations was observed (Castillo et al., 2020). The results of the pairwise comparisons between the window durations can be found in Table S1. Table 3 shows that the median accuracy decreased from 0.95 for the 1-s window to 0.79 for the 7-s window, indicating that more segments were classified correctly with a shorter window.

**Table 3:** Summary of statistics-based validation results. The table includes: the window duration (in seconds), the number of EEG recordings across which the median was calculated (*N*); the median accuracy; the median hit rate (HR); the median false discovery rate (FDR); the median of the product between accuracy and HR (Acc*HR). For each metric, the best median values are marked in bold and the median values that are statistically different from the best value are marked with an asterisk (*).

Window duration (s)	*N*	Accuracy	HR	FDR	Acc*HR
1	16	**0.95**	0.83*	**0.18**	0.76
2	16	0.93	0.88	0.30	0.79
3	16	0.91	0.91	0.37	**0.81**
4	16	0.86	0.95	0.49	0.80
5	16	0.83*	0.96	0.54	0.77
6	16	0.81*	0.95	0.55*	0.75
7	16	0.79*	**0.98**	0.60*	0.74

The effect size of HR ( ${\chi ^2} = 20.15$, degrees of freedom = 111, $p = 0.0026$, $E_R^2 = 0.18$) indicated a relatively strong difference in HR between the different window durations (Castillo et al., 2020). The results of the pairwise comparisons between the window durations can be found in Table S2. The median HR (Table 3) increased from 0.83 for the 1-s window to 0.98 for the 7-s window, but it reached a plateau from the 4-s window onwards. The increase of the median HR from windows of 1 s to windows of 4 s suggests that the algorithm detects more annotated artefacts (*i.e*., a larger number of true positives) when longer windows are used.

Equivalent to HR, a relatively strong difference in FDR between the different window durations was observed ( ${\chi ^2} = 19.64$, degrees of freedom = 111, $p = 0.0032$, $E_R^2 = 0.18$) (Castillo et al., 2020). The results of the pairwise comparisons between the window durations can be found in Table S3. The median FDR (Table 3) showed the same trend as the median HR, increasing from 0.18 to 0.60 with increasing window durations. However, this measure should be as close to zero as possible. Hence, the use of larger windows would lead to an increase of false positives.

To identify the best window for the automated algorithm, we considered that the accuracy and HR should be as high as possible whereas the FDR should be as small as possible. For each patient of Dataset 3 and each window duration, the product of accuracy and HR was calculated to identify the window duration(s) with the best combination of these metrics (Table 3). No significant differences between the different window durations were observed after applying a Kruskal-Wallis test with Bonferroni correction for pairwise comparisons. The results of the pairwise comparisons between the window durations are given in Table S4.

Table 3 shows the median values of the statistical metrics for the different window durations. The window of 3 s duration had the best combination of accuracy and HR (Accuracy = 0.91; HR = 0.91), immediately followed by the windows of 2 and 4 s duration (2-s window: Accuracy = 0.93; HR = 0.88; 4-s window: Accuracy = 0.86; HR = 0.95). If we then look at the values of FDR, the window of 1 s duration had the best FDR and the best accuracy (FDR = 0.18, Accuracy = 0.95), but the worst HR (HR = 0.83), which was significantly lower than the highest HR, obtained for a window of 7 s. Also based on the quite high value of FDR, we decided not to consider the 4-s window in the application-based validation of our method. Consequently, we selected the windows with 2 and 3 s duration for validating the algorithm with the four-stage sleep classifier.

An overview of the results obtained for the 20-min segments of the EEG recording of the 16 infants of Dataset 3 when using a window duration of 2 and 3 s can be found in Table S5. Generally, the percentage of the signal that was removed increased for longer windows, but the removal percentage ranged from 4.58% to 49.25% for the 2-s window and from 7.00% to 52.08% for the 3-s window across the sixteen EEG recordings and was always below 40.00% except for two EEG recordings (*i.e*., infant 4 and 6).

### Application-based validation

Table 4 shows the results for the Wilcoxon rank sum test comparing the accuracy, mean precision, mean sensitivity and mean confidence of the four-stage sleep classifier before and after applying the proposed algorithm using the 2- and 3-s windows. A relatively strong difference in accuracy (2-s window: +10.63%, ${\eta ^2} = 0.22$; 3-s window: +11.17%, ${\eta ^2} = 0.25$) and mean confidence (2-s window: +7.68%, ${\eta ^2} = 0.28$; 3-s window: +7.73%, ${\eta ^2} = 0.29$), and a moderate difference in mean precision (2-s window: +9.38%, ${\eta ^2} = 0.12$; 3-s window: +9.83%, ${\eta ^2} = 0.13$) was observed for both the 2- and 3-s window, with better values after removal of flat line segments and large amplitude fluctuations from the EEG signals. On the other hand, no significant difference was observed for the mean sensitivity after artefacts removal (2-s window: +0.83%, ${\eta ^2} = 0.0009$; 3-s window: +2.25%, ${\eta ^2} = 0.0059$). When comparing the two window durations, it was seen that the accuracy, mean precision and mean sensitivity were always slightly higher when a 3-s window was used, whereas no clear differences in mean confidence could be observed.

**Table 4:** Wilcoxon rank sum test results. The table compares the accuracy, mean precision, mean sensitivity and mean confidence of the four-stage sleep classifier before and after removing, by applying our proposed algorithm, flat line segments (FL) and large amplitude fluctuations (LA) with windows of 2 and 3 s duration. The table includes: the number of observations (*i.e*., the sixteen EEG recordings present in Dataset 3) (*N*); the median of the considered statistical measure; the rank sum test statistic; the Z-statistic; the corresponding *p*-value and the effect size. The best median value of each statistical measure and the significant differences (*i.e*., *p*-value ≤ 0.05) are marked in bold.

Statistical measure	Window duration (s)	Before/after FL & LA removal	*N*	Median	Rank sum test statistic	Z-statistic	*p*-value	${\bi{\eta }^2}$
Accuracy	2	Before	16	39.05	335	2.66	**0.0079**	0.22
		After	16	49.68				
	3	Before	16	39.05	340	2.85	**0.0044**	0.25
		After	16	**50.22**				
Mean precision	2	Before	16	44.45	317	1.98	**0.05**	0.12
		After	16	53.83				
	3	Before	16	44.45	319	2.05	**0.04**	0.13
		After	16	**54.28**				
Mean sensitivity	2	Before	16	72.36	269	0.17	0.87	0.0009
		After	16	73.19				
	3	Before	16	72.36	276	0.43	0.66	0.0059
		After	16	**74.61**				
Mean confidence	2	Before	16	35.13	344	3.00	**0.0027**	0.28
		After	16	42.81				
	3	Before	16	35.13	345	3.03	**0.0024**	0.29
		After	16	**42.86**				

## Discussion

An algorithm for the automated detection and removal of flat line segments and large amplitude fluctuations from neonatal EEG signals of infants at term-equivalent age was developed. The proposed algorithm was tested and validated on EEG data recorded with different EEG systems and with caps mounting a different number of electrodes in order to make the algorithm as general as possible. The first issue to solve was the identification of the window duration that would lead to an optimal performance of the algorithm. In that regard, the results for the different performance metrics were contradictory to each other, as the median accuracy and median FDR suggested that using windows of 1 s duration would yield to the best algorithm performance, whereas the best median HR was obtained for windows of 7 s duration. Consequently, there will always be a trade-off between deleting enough artefactual segments but retaining a sufficient number of non-artefactual EEG segments to preserve cortical information. Considering this trade-off, it would be recommended to use a window duration of 2 or 3 s in the algorithm because their products of the accuracy and HR were the highest compared to the products of the other window durations. In addition, their accuracy, HR and FDR did not significantly differ from those of the window durations with the highest value for each of these performance metrics.

When windows of 2 or 3 s duration were used, the obtained FDRs indicated the presence of a high number of false positives. Nevertheless, given the fact that the percentages of removed EEG segments were almost always below 40% of the EEG recording (see Table S5) and that the EEG recordings were often hours long, these high FDRs are acceptable. This result might induce to think that correcting artefacts would be better than removing them, but flat line segments cannot be corrected because no cortical activity was recorded during those intervals. Large amplitude fluctuations are very difficult to correct given the high variability of their waveform and peak-to-peak amplitude. Moreover, not only one channel is generally affected by this artefact. Corrections performed, *e.g*., by interpolation of the neighboring channels are at risk of considering as brain activity a reconstructed signal that is in fact strongly affected by an artefact.

When comparing our results with those of previous studies which detected flat line segments and large amplitude fluctuations within a wider range of artefacts, our proposed algorithm performed equally well or even better. For instance, Costa (2017) reported a true positive rate of 92.4% when detecting movement/electrode displacement artefacts and Kauppila, Vanhatalo & Stevenson (2017) obtained a median accuracy of 85.7% and 97.7% for movement and flat artefacts, respectively. Also, Webb et al. (2021) reported an accuracy of 80.7% and 43.9% when detecting movement artefacts on the training and on the validation data, respectively.

The proposed algorithm was further validated by comparing the performance of a four-stage sleep classifier (Ansari et al., 2020) before and after applying the proposed algorithm using the window durations that were identified as yielding the best algorithm performance, *i.e*., those with 2 and 3 s duration. The confidence of the sleep classifier resulted to be significantly higher when flat line segments and large amplitude fluctuations were removed compared to when they were still present in the EEG signal. Likewise, the accuracy and mean precision of the classifier were significantly higher after applying the proposed artefact removal method. Conversely, no significant difference was observed in the mean sensitivity of the classifier, which is in line with a similar study by Khlif et al. (2010) who found an increase of only 0.1% in the sensitivity, but a 18.3% increase in the accuracy of a seizure detector after artefact reduction. Also De Vos et al. (2011) observed an improvement in seizure detection after artefact removal, with a significant decrease in median false alarms per hour from 0.38 to 0.00 and a non-significant increase in median good detection rate from 87.1% to 100.0%. Similarly to our study, Dereymaeker et al. (2017) investigated the improvement in performance of a sleep classifier and reached a 13% increase in the median accuracy which is comparable to the 10.63–11.17% increase observed in our study. Differently from us, Dereymaeker et al. (2017) also reported an increase of 16% in the median sensitivity.

Taking a closer look at the different performance measures of the four-stage sleep classifier between the two window durations, a window of 3 s duration showed slightly higher accuracy, mean precision, mean sensitivity and effect sizes than the 2-s window. Therefore, a 3-s window would be recommended for this EEG application. In conclusion, this validation demonstrated the effectiveness of the proposed automated artefact detection and removal method in neonatal EEG application and confirmed the good performance of the algorithm.

A few shortcomings should be addressed in future research. Firstly, the proposed algorithm was tuned to be applied to EEG signals of infants at term-equivalent age. Consequently, the algorithm and more specifically the physiological threshold will need adjustment when applied to EEG signals of infants with a younger age (PMA < 36 weeks) because physiological amplitudes increase with decreasing age (André et al., 2010). Secondly, the proposed algorithm was tested only in healthy, non-pathological EEG signals. Therefore, the current detection criteria might not hold when applied to EEG signals where–for instance–seizures are present. For this reason, future studies should aim at testing the algorithm on pathological EEG signals and adjusting it accordingly. Also, it should be noted that the proposed algorithm detects and removes only flat line segments and large amplitude fluctuations. Additional artefacts affecting the neonatal EEG recordings, such as muscular artefact, cardiac interference, and eye movements should be detected and/or removed by means of other dedicated methods to be developed. Finally, the ultimate choice between using a window of 2 or 3 s and the thresholds could need to be adjusted to the specific features of the EEG recording to which the algorithm should be applied, such as its sampling frequency and the recording length.

## Conclusions

We described an algorithm for the automated detection and removal of flat line segments and large amplitude fluctuations in neonatal EEG recordings. The algorithm achieved a median accuracy and HR comparable to or higher than what achieved by other similar methods. The quite high value of the median FDR obtained for windows equal or longer than 3 s, should be considered in association with the very good values of accuracy and HR, which were very high. Similarly to other authors, we also validated the effectiveness of the algorithm by applying it to the neonatal EEG signals of Dataset 3 and assessing the performance of a four-stage sleep classifier after having applied our algorithm. We could verify that the performance of the classifier improved by about 10% in terms of accuracy, mean confidence and mean precision with respect to its application without having priorly applied our algorithm. This result shows that our algorithm has a good performance and can be effective for neonatal EEG applications.

Our algorithm also shows some general advantages with respect to existing methods to detect artefacts on the neonatal EEG: (1) the thresholds used for detecting flat lines and large amplitude fluctuations were tailored to the term-equivalent age of the neonate because it has an influence on the amplitude of the EEG signals; (2) the proposed algorithm detects flat line segments and large amplitude fluctuations with an easy approach that has a low computational load, hence prone to be easily used in the clinical practice; (3) the performance of the algorithm was assessed by calculating statistical measures, and not only by validating it in an analytical application (the sleep classifier) as most existing methods do.

## Supplemental Information

10.7717/peerj.13734/supp-1 Supplemental Information 1 Results of the pairwise comparisons of the accuracy of the algorithm using the different window durations. We used the Kruskal-Wallis test after Bonferroni correction on Dataset 3. The table includes: the two window durations that are compared; the difference in mean rank of the accuracy between the two considered window durations; the lower and upper limits of the 95% confidence interval of the mean rank difference and corresponding *p*-value with null hypothesis that mean rank difference is equal to zero (significant differences in mean rank are indicated in bold, *i.e*., *p*-value ≤ 0.05).

10.7717/peerj.13734/supp-2 Supplemental Information 2 Results of the pairwise comparisons of the hit rate (HR) of the algorithm using the different window durations. We used the Kruskal-Wallis test after Bonferroni correction on Dataset 3. The table includes: the two window durations that are compared; the difference in mean rank of the hit rate (HR) between the two considered window durations; the lower and upper limits of the 95% confidence interval of the mean rank difference and corresponding *p*-value with null hypothesis that mean rank difference is equal to zero (significant differences in mean rank are indicated in bold, *i.e*., *p*-value ≤ 0.05).

10.7717/peerj.13734/supp-3 Supplemental Information 3 Results of the pairwise comparisons of the false discovery rate (FDR) of the algorithm using the different window durations. We used the Kruskal-Wallis test after Bonferroni correction on Dataset 3. The table includes: the two window durations that are compared; the difference in mean rank of the false discovery rate between the two considered window durations; the lower and upper limits of the 95% confidence interval of the mean rank difference and corresponding *p*-value with null hypothesis that mean rank difference is equal to zero (significant differences in mean rank are indicated in bold, *i.e*., *p*-value ≤ 0.05).

10.7717/peerj.13734/supp-4 Supplemental Information 4 Results of the pairwise comparisons of the product of the accuracy and hit rate (HR) of the algorithm using different window durations. We used the Kruskal-Wallis test after Bonferroni correction on Dataset 3. The table includes: the two window durations that are compared; the difference in mean rank of the product of accuracy and HR between the two considered window durations; the lower and upper limits of the 95% confidence interval of the mean rank difference and the corresponding *p*-value with null hypothesis that mean rank difference is equal to zero.

10.7717/peerj.13734/supp-5 Supplemental Information 5 Results for the 20-min segments of the EEG recordings of the 16 infants of Dataset 3. The table includes: the percentage of the signal that was annotated as large amplitude fluctuations (% annotations), the window duration (in seconds), the percentage of the signal that was detected as large amplitude fluctuations and removed by the algorithm (% removed), the accuracy, the hit rate (HR) and the false discovery rate (FDR) of the algorithm. The average for each measure per window duration is given at the bottom of the table.

## Additional Information and Declarations

### Data Availability

The following information was supplied regarding data availability: The EEG recordings included in Dataset 1 used in this study are available at Zenodo: Nathan Stevenson, Karoliina Tapani, Leena Lauronen, & Sampsa Vanhatalo. (2019). A dataset of neonatal EEG recordings with seizures annotations [Data set]. Zenodo. https://doi.org/10.5281/zenodo.2547147.
