# Supplementary material for: Automated detection and removal of flat line segments and large amplitude fluctuations in neonatal electroencephalography
Source: PeerJ. 2022 Jul 12;10:e13734. doi: 10.7717/peerj.13734 (PMC9285485; doi:10.7717/peerj.13734)
Supplement: Supplemental Information 1 — We used the Kruskal-Wallis test after Bonferroni correction on Dataset 3. The table includes: the two window durations that are compared; the difference in mean rank of the accuracy between the two considered window durations; the lower and upper limits of the 95% confidence interval of the mean rank difference and corresponding p-value with null hypothesis that mean rank difference is equal to zero (significant differences in mean rank are indicated in bold, i.e., p-value ≤ 0.05). [file peerj-10-13734-s001.docx]

| Window duration 1 (s) | Window duration 2 (s) | Mean rank difference | 95% Confidence interval | | p-value |
| --- | --- | --- | --- | --- | --- |
|  |  |  | **Lower limit** | **Upper limit** |  |
| 1 | 2 | 10.22 | -24.62 | 45.05 | 1.00 |
| 1 | 3 | 18.00 | -16.83 | 52.83 | 1.00 |
| 1 | 4 | 30.31 | -4.52 | 65.15 | 0.17 |
| 1 | 5 | 42.19 | 7.35 | 77.02 | **0.0049** |
| 1 | 6 | 52.13 | 17.29 | 86.96 | **0.0001** |
| 1 | 7 | 56.50 | 21.67 | 91.33 | **1.75** $\boldsymbol{\cdot}$ **10^-5^** |
| 2 | 3 | 7.78 | -27.05 | 42.62 | 1.00 |
| 2 | 4 | 20.09 | -14.74 | 54.93 | 1.00 |
| 2 | 5 | 31.97 | -2.87 | 66.80 | 0.11 |
| 2 | 6 | 41.91 | 7.07 | 76.74 | **0.0054** |
| 2 | 7 | 46.28 | 11.45 | 81.12 | **0.0011** |
| 3 | 4 | 12.31 | -22.52 | 47.15 | 1.00 |
| 3 | 5 | 24.19 | -10.65 | 59.02 | 0.73 |
| 3 | 6 | 34.13 | -0.71 | 68.96 | 0.06 |
| 3 | 7 | 38.50 | 3.67 | 73.33 | **0.02** |
| 4 | 5 | 11.88 | -22.96 | 46.71 | 1.00 |
| 4 | 6 | 21.81 | -13.02 | 56.65 | 1.00 |
| 4 | 7 | 26.19 | -8.65 | 61.02 | 0.47 |
| 5 | 6 | 9.94 | -24.90 | 44.77 | 1.00 |
| 5 | 7 | 14.31 | -20.52 | 49.15 | 1.00 |
| 6 | 7 | 4.38 | -30.46 | 39.21 | 1.00 |
