# Supplementary material for: Automated detection and removal of flat line segments and large amplitude fluctuations in neonatal electroencephalography
Source: PeerJ. 2022 Jul 12;10:e13734. doi: 10.7717/peerj.13734 (PMC9285485; doi:10.7717/peerj.13734)
Supplement: Supplemental Information 2 — We used the Kruskal-Wallis test after Bonferroni correction on Dataset 3. The table includes: the two window durations that are compared; the difference in mean rank of the hit rate (HR) between the two considered window durations; the lower and upper limits of the 95% confidence interval of the mean rank difference and corresponding p-value with null hypothesis that mean rank difference is equal to zero (significant differences in mean rank are indicated in bold, i.e., p-value ≤ 0.05). [file peerj-10-13734-s002.docx]

| Window duration 1 (s) | Window duration 2 (s) | Mean rank difference | 95% Confidence interval | | p-value |
| --- | --- | --- | --- | --- | --- |
|  |  |  | **Lower limit** | **Upper limit** |  |
| 1 | 2 | -8.06 | -42.89 | 26.76 | 1.00 |
| 1 | 3 | -19.69 | -54.51 | 15.14 | 1.00 |
| 1 | 4 | -29.28 | -64.10 | 5.54 | 0.22 |
| 1 | 5 | -35.56 | -70.39 | -0.74 | **0.04** |
| 1 | 6 | -32.59 | -67.42 | 2.23 | 0.09 |
| 1 | 7 | -39.53 | -74.35 | -4.71 | **0.01** |
| 2 | 3 | -11.63 | -46.45 | 23.20 | 1.00 |
| 2 | 4 | -21.22 | -56.04 | 13.60 | 1.00 |
| 2 | 5 | -27.50 | -62.32 | 7.32 | 0.35 |
| 2 | 6 | -24.53 | -59.35 | 10.29 | 0.68 |
| 2 | 7 | -31.47 | -66.29 | 3.35 | 0.13 |
| 3 | 4 | -9.59 | -44.42 | 25.23 | 1.00 |
| 3 | 5 | -15.88 | -50.70 | 18.95 | 1.00 |
| 3 | 6 | -12.91 | -47.73 | 21.92 | 1.00 |
| 3 | 7 | -19.84 | -54.67 | 14.98 | 1.00 |
| 4 | 5 | -6.28 | -41.10 | 28.54 | 1.00 |
| 4 | 6 | -3.31 | -38.14 | 31.51 | 1.00 |
| 4 | 7 | -10.25 | -45.07 | 24.57 | 1.00 |
| 5 | 6 | 2.97 | -31.85 | 37.79 | 1.00 |
| 5 | 7 | -3.97 | -38.79 | 30.85 | 1.00 |
| 6 | 7 | -6.94 | -41.76 | 27.89 | 1.00 |
