# Supplementary material for: Automated detection and removal of flat line segments and large amplitude fluctuations in neonatal electroencephalography
Source: PeerJ. 2022 Jul 12;10:e13734. doi: 10.7717/peerj.13734 (PMC9285485; doi:10.7717/peerj.13734)
Supplement: Supplemental Information 3 — We used the Kruskal-Wallis test after Bonferroni correction on Dataset 3. The table includes: the two window durations that are compared; the difference in mean rank of the false discovery rate between the two considered window durations; the lower and upper limits of the 95% confidence interval of the mean rank difference and corresponding p-value with null hypothesis that mean rank difference is equal to zero (significant differences in mean rank are indicated in bold, i.e., p-value ≤ 0.05). [file peerj-10-13734-s003.docx]

| Window duration 1 (s) | Window duration 2 (s) | Mean rank difference | 95% Confidence interval | | p-value |
| --- | --- | --- | --- | --- | --- |
|  |  |  | **Lower limit** | **Upper limit** |  |
| 1 | 2 | -13.50 | -48.38 | 21.38 | 1.00 |
| 1 | 3 | -20.31 | -55.19 | 14.56 | 1.00 |
| 1 | 4 | -27.69 | -62.56 | 7.19 | 0.33 |
| 1 | 5 | -34.56 | -69.44 | 0.31 | 0.05 |
| 1 | 6 | -38.00 | -72.88 | -3.12 | **0.02** |
| 1 | 7 | -40.72 | -75.59 | -5.84 | **0.0082** |
| 2 | 3 | -6.81 | -41.69 | 28.06 | 1.00 |
| 2 | 4 | -14.19 | -49.06 | 20.69 | 1.00 |
| 2 | 5 | -21.06 | -55.94 | 13.81 | 1.00 |
| 2 | 6 | -24.50 | -59.38 | 10.38 | 0.69 |
| 2 | 7 | -27.22 | -62.09 | 7.66 | 0.37 |
| 3 | 4 | -7.38 | -42.25 | 27.50 | 1.00 |
| 3 | 5 | -14.25 | -49.13 | 20.63 | 1.00 |
| 3 | 6 | -17.69 | -52.56 | 17.19 | 1.00 |
| 3 | 7 | -20.41 | -55.28 | 14.47 | 1.00 |
| 4 | 5 | -6.88 | -41.75 | 28.00 | 1.00 |
| 4 | 6 | -10.31 | -45.19 | 24.56 | 1.00 |
| 4 | 7 | -13.03 | -47.91 | 21.84 | 1.00 |
| 5 | 6 | -3.44 | -38.31 | 31.44 | 1.00 |
| 5 | 7 | -6.16 | -41.03 | 28.72 | 1.00 |
| 6 | 7 | -2.72 | -37.59 | 32.16 | 1.00 |
