# Supplementary material for: Automated detection and removal of flat line segments and large amplitude fluctuations in neonatal electroencephalography
Source: PeerJ. 2022 Jul 12;10:e13734. doi: 10.7717/peerj.13734 (PMC9285485; doi:10.7717/peerj.13734)
Supplement: Supplemental Information 4 — We used the Kruskal-Wallis test after Bonferroni correction on Dataset 3. The table includes: the two window durations that are compared; the difference in mean rank of the product of accuracy and HR between the two considered window durations; the lower and upper limits of the 95% confidence interval of the mean rank difference and the corresponding p-value with null hypothesis that mean rank difference is equal to zero. [file peerj-10-13734-s004.docx]

| Window duration 1 (s) | Window duration 2 (s) | Mean rank difference | 95% Confidence interval | | p-value |
| --- | --- | --- | --- | --- | --- |
|  |  |  | **Lower limit** | **Upper limit** |  |
| 1 | 2 | -4.50 | -39.38 | 30.38 | 1.00 |
| 1 | 3 | -7.97 | -42.85 | 26.91 | 1.00 |
| 1 | 4 | -8.16 | -43.04 | 26.73 | 1.00 |
| 1 | 5 | -3.09 | -37.98 | 31.79 | 1.00 |
| 1 | 6 | 5.03 | -29.85 | 39.91 | 1.00 |
| 1 | 7 | 6.44 | -28.44 | 41.32 | 1.00 |
| 2 | 3 | -3.47 | -38.35 | 31.41 | 1.00 |
| 2 | 4 | -3.66 | -38.54 | 31.23 | 1.00 |
| 2 | 5 | 1.41 | -33.48 | 36.29 | 1.00 |
| 2 | 6 | 9.53 | -25.35 | 44.41 | 1.00 |
| 2 | 7 | 10.94 | -23.94 | 45.82 | 1.00 |
| 3 | 4 | -0.19 | -35.07 | 34.69 | 1.00 |
| 3 | 5 | 4.88 | -30.01 | 39.76 | 1.00 |
| 3 | 6 | 13.00 | -21.88 | 47.88 | 1.00 |
| 3 | 7 | 14.41 | -20.48 | 49.29 | 1.00 |
| 4 | 5 | 5.06 | -29.82 | 39.94 | 1.00 |
| 4 | 6 | 13.19 | -21.69 | 48.07 | 1.00 |
| 4 | 7 | 14.59 | -20.29 | 49.48 | 1.00 |
| 5 | 6 | 8.13 | -26.76 | 43.01 | 1.00 |
| 5 | 7 | 9.53 | -25.35 | 44.41 | 1.00 |
| 6 | 7 | 1.41 | -33.48 | 36.29 | 1.00 |
