# Supplementary material for: Automated detection and removal of flat line segments and large amplitude fluctuations in neonatal electroencephalography
Source: PeerJ. 2022 Jul 12;10:e13734. doi: 10.7717/peerj.13734 (PMC9285485; doi:10.7717/peerj.13734)
Supplement: Supplemental Information 5 — The table includes: the percentage of the signal that was annotated as large amplitude fluctuations (% annotations), the window duration (in seconds), the percentage of the signal that was detected as large amplitude fluctuations and removed by the algorithm (% removed), the accuracy, the hit rate (HR) and the false discovery rate (FDR) of the algorithm. The average for each measure per window duration is given at the bottom of the table. [file peerj-10-13734-s005.docx]

| Infant nr. | % annotations | Window duration (s) | % removed | Accuracy | HR | FDR |
| --- | --- | --- | --- | --- | --- | --- |
| 1 | 27.17 | 2 | 28.50 | 0.86 | 0.77 | 0.27 |
|  |  | 3 | 31.00 | 0.83 | 0.76 | 0.33 |
| 2 | 13.58 | 2 | 18.58 | 0.92 | 0.87 | 0.36 |
|  |  | 3 | 21.17 | 0.91 | 0.93 | 0.40 |
| 3 | 31.58 | 2 | 32.67 | 0.92 | 0.89 | 0.14 |
|  |  | 3 | 34.50 | 0.92 | 0.92 | 0.15 |
| 4 | 48.25 | 2 | 49.25 | 0.95 | 0.96 | 0.06 |
|  |  | 3 | 52.08 | 0.94 | 0.98 | 0.09 |
| 5 | 17.50 | 2 | 22.92 | 0.93 | 0.95 | 0.27 |
|  |  | 3 | 24.75 | 0.92 | 0.98 | 0.31 |
| 6 | 19.42 | 2 | 40.42 | 0.76 | 0.93 | 0.55 |
|  |  | 3 | 50.83 | 0.68 | 1.00 | 0.62 |
| 7 | 4.50 | 2 | 7.92 | 0.96 | 0.98 | 0.44 |
|  |  | 3 | 10.75 | 0.94 | 1.00 | 0.58 |
| 8 | 31.00 | 2 | 25.58 | 0.84 | 0.66 | 0.20 |
|  |  | 3 | 26.33 | 0.84 | 0.67 | 0.21 |
| 9 | 16.33 | 2 | 19.33 | 0.93 | 0.88 | 0.25 |
|  |  | 3 | 21.17 | 0.92 | 0.89 | 0.31 |
| 10 | 22.00 | 2 | 24.58 | 0.94 | 0.92 | 0.18 |
|  |  | 3 | 25.67 | 0.94 | 0.94 | 0.19 |
| 11 | 2.83 | 2 | 4.58 | 0.94 | 0.29 | 0.82 |
|  |  | 3 | 7.00 | 0.92 | 0.35 | 0.86 |
| 12 | 15.08 | 2 | 21.83 | 0.89 | 0.87 | 0.40 |
|  |  | 3 | 27.50 | 0.85 | 0.90 | 0.51 |
| 13 | 50.83 | 2 | 35.83 | 0.76 | 0.62 | 0.12 |
|  |  | 3 | 38.00 | 0.79 | 0.66 | 0.11 |
| 14 | 12.92 | 2 | 16.92 | 0.93 | 0.88 | 0.33 |
|  |  | 3 | 20.75 | 0.90 | 0.90 | 0.44 |
| 15 | 4.92 | 2 | 10.00 | 0.93 | 0.78 | 0.62 |
|  |  | 3 | 12.00 | 0.91 | 0.81 | 0.67 |
| 16 | 8.92 | 2 | 17.50 | 0.89 | 0.85 | 0.57 |
|  |  | 3 | 20.75 | 0.87 | 0.94 | 0.59 |
| AVERAGE | 20.43 | 2 | 23.53 | 0.90 | 0.82 | 0.35 |
|  |  | 3 | 26.52 | 0.88 | 0.85 | 0.40 |
